# Deep Learning-Based Studies on Pediatric Brain Tumors Imaging: Narrative Review of Techniques and Challenges

**DOI:** 10.3390/brainsci11060716

**Published:** 2021-05-28

**Authors:** Hala Shaari, Jasmin Kevrić, Samed Jukić, Larisa Bešić, Dejan Jokić, Nuredin Ahmed, Vladimir Rajs

**Affiliations:** 1 Department of Information Technologies, Faculty of Engineering and Natural Sciences, International BURCH University, 71000 Sarajevo, Bosnia and Herzegovina; hala.shaari@stu.ibu.edu.ba; 2 Faculty of Engineering and Natural Sciences, International BURCH University, 71000 Sarajevo, Bosnia and Herzegovina; jasmin.kevric@ibu.edu.ba (J.K.); Samed.Jukic@ibu.edu.ba (S.J.); larisa.besic@ibu.edu.ba (L.B.); dejan.jokic@ibu.edu.ba (D.J.); 3 Control Department, Technical Computer College Tripoli, Tripoli 00218, Libya; nuredin@mtit.com.ly; 4 Department of Power, Electronics and Telecommunication Engineering, Faculty of Technical Science, University of Novi Sad, 21000 Novi Sad, Serbia

**Keywords:** deep learning, pediatric brain tumor, children tumor, medical images

## Abstract

Brain tumors diagnosis in children is a scientific concern due to rapid anatomical, metabolic, and functional changes arising in the brain and non-specific or conflicting imaging results. Pediatric brain tumors diagnosis is typically centralized in clinical practice on the basis of diagnostic clues such as, child age, tumor location and incidence, clinical history, and imaging (Magnetic resonance imaging MRI / computed tomography CT) findings. The implementation of deep learning has rapidly propagated in almost every field in recent years, particularly in the medical images’ evaluation. This review would only address critical deep learning issues specific to pediatric brain tumor imaging research in view of the vast spectrum of other applications of deep learning. The purpose of this review paper is to include a detailed summary by first providing a succinct guide to the types of pediatric brain tumors and pediatric brain tumor imaging techniques. Then, we will present the research carried out by summarizing the scientific contributions to the field of pediatric brain tumor imaging processing and analysis. Finally, to establish open research issues and guidance for potential study in this emerging area, the medical and technical limitations of the deep learning-based approach were included.

## 1. Introduction

The second most common pediatric tumors in childhood after leukemia are nervous system tumors. Brain cancer is cancer that usually occurs in children, account for about 15% of pediatric cancers, sometime between birth and the age of 14 years. Brain tumors may be classified according to their source or aggression, with primary brain tumors arising in the brain, while metastatic brain tumors may occur in other parts of the body. The Health Organization (WHO), which classifies brain tumors with increasing aggressiveness in Grades I to IV, launched the most widely used grading classification scheme in 1993 [1]. 

This classification is depending on region, type of tissue, degree of malignancy and various other factors. After malignancy level determinations of microscopic tested tumor cells, its grade can be assessed using cell growth rate, cells blood supply, centered dead tumor cells, and tumor cell resemblance to normal cells. The most common cancers in the pediatric age group include glioma, ependymoma, medulloblastoma, craniopharyngioma, and pinealoma. Infratentorial and supratentorial tumors arise at around the same level in infants; tumors of germ cells, teratomas, gliomas, neuroepithelial tumors (PNETs) and papillomas of the choroid plexus are recognized. Whereas, posterior fossa neoplasms (primitive neuroepithelial tumors, ependymomas, astrocytomas and hemangioblastomas) are often found in older children.

Since each type of tumor can be given special treatment, radiotherapy, surgery, chemotherapy, are some of the therapeutic options available [2,3]. However, before any treatment is given, it is important to consider the nature of the brain tumor, including its size, rate of growth, and all the contributing factors mentioned earlier. Nowadays, histological and molecular diagnosis of tumors is certainly the most important thing to consider in order to understand prognosis, therapy and survival. In pediatric patients, tumor samples are also regularly subjected to genetic and protein fusion testing in addition to traditional histologic tests, providing a new degree of diagnostic accuracy. Additionally, a more precise prognosis could be possible for outcome studies that take into account molecular subgrouping. This knowledge would certainly aid us in tailoring treatment regimens for different tumor subgroups [4,5].

Magnetic resonance imaging MRI, is the standard imaging technique for the diagnosis of brain tumors [6,7]. As a non-invasive technique that is widely available in clinics, MRI provides excellent contrast between soft tissues [8]. MRI is used to provide the most accurate tumor pathology and metabolism data in combination with other imaging methods, such as computed tomography and magnetic resonance spectroscopy.

The aim of this review is to provide a detailed summary of the current state of pediatric brain tumors studies centered on medical imaging based on deep learning. The remaining of this review study is arranged as follows: Section 2 introduces briefly pediatric brain tumor types, defines pediatric brain imaging techniques and explores the different MRI sequences to provide an inclusive background on the field. This is followed by available datasets for pediatric brain tumor modalities and data acquisition and analysis methods for human brain activity. Section 3 presents the research carried out by summarizing the scientific contributions to the field of pediatric brain tumor imaging processing and analysis. Medical and technical challenges in pediatric brain tumor can be found in Section 4. The conclusion and research directions are provided in Section 5.

## 2. Related Works

### 2.1. Brain Tumor in Childhood

**Figure 1:**
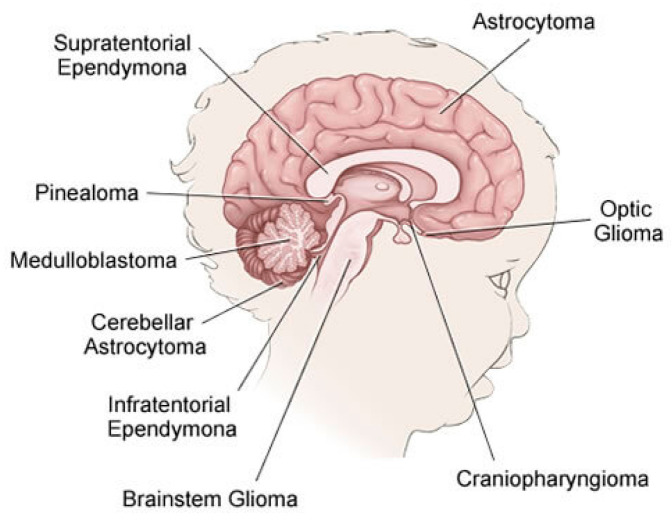
Types of the CNS Tumors in Children.

**Figure 2:**
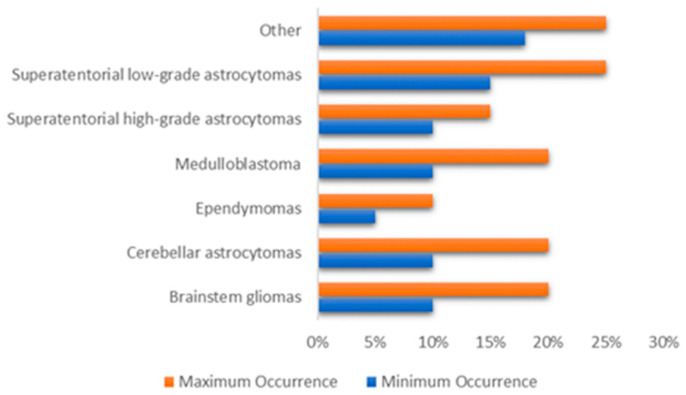
The approximate occurrence of common pediatric brain tumors.

The brain is primarily divided into three parts: the cerebrum, cerebellum, and brain stem, which make up the central nervous system along with the spinal cord (CNS). Tumors may form in almost any type of brain or spinal cord tissue or cell. However certain tumors can contain a combination of various types of cells. Different forms of tumors appear to originate in certain areas of the brain and start to grow in certain ways as can be shown in Figure 1 [9] and the approximate occurrence of common pediatric brain tumors is also demonstrated in Figure 2 [10]. According to the American Cancer Society [11], the most prevalent forms of central nervous system (CNS) tumors in children are:

1. Gliomas: Is a generic name for a number of cancers, including:
  - Astrocytomas (which include glioblastomas): From a specific type of glial cells called astrocytes, these types of tumors are usually started. They are often grouped by grade. Low-grade astrocytomas include pilocytic astrocytomas, subependymal giant cell astrocytomas (SEGAs), diffuse astrocytomas, pleomorphic xanthoastrocytomas (PXAs) and optic gliomas. High-grade astrocytomas include glioblastomas and anaplastic astrocytomas.
  - Oligodendrogliomas: From a specific type of cerebral cells called oligodendrocytes, these types of tumor are usually started. Oligodendrogliomas have been categorized as Grade II tumors that account for over 1% of children’s brain tumors.
  - Ependymomas: From the ependymal cells which line the spinal cord, begins this type of tumors which responsible for around 5% of brain tumors in children. They can vary from Grade I tumors to Grade III tumors (anaplastic ependymomas).
  - Brainstem gliomas: This tumor is a glioma that develops in brain stem and is responsible for around 10% to 20% of brain tumors in children. They are common in two types: focal brain stem gliomas or diffuse midline gliomas.
2. Embryonal tumors: These tumors begin in the central nervous system, in early forms of nerve cells. In children, they are common among younger children rather than older children. Embryonic tumors account for around 10–20% of brain tumors, including the most frequent type: medulloblastomas, and less common types such as medulloepithelioma, and atypical teratoid (ATRT).
3. Pineal tumors: Are there any types of tumors that could be found in the pineal gland? The most popular, fastest growing and difficult to treat type of these forms is called pineoblastomas.
4. Craniopharyngiomas: Craniopharyngiomas account for approximately 4% of children’s brain tumors. They occur over the pituitary gland, but it is under the brain itself that these slow-growing tumors begin.
5. Mixed neuronal and glial tumors: This type of tumor combined between neuronal and glial tumors. They include dysembryoplastic neuroepithelial tumors (DNETs) and gangliogliomais.
6. Choroid plexus tumors: They are a rare tumor, many of which are benign and some are malignant.
7. Schwannomas: They begin in cells that surround and separate the cranial nerves and other nerves. These rare tumors are usually benign.
8. In or near brain tumors: These include chordomas, tumors of germ cells, neuroblastomas, pituitary tumors, meningiomas (Grade I to Grade III) and lymphomas.
9. Metastatic or secondary brain tumors: The tumors that begin in other organs and then spread to the brain are metastatic or secondary brain tumors. They are often less frequent than primary brain tumors and often treated differently.

### 2.2. Pediatric Brain Imaging Technique

The primarily used imaging techniques include MRI (magnetic resonance imaging) and its various applications, such as MR spectroscopy, MR perfusion and functional MRI along with CT (computed tomography), and PET (positron emission tomography). Many of these techniques use agents, such as gadolinium, which improve contrast. In addition to these techniques, there are also other diagnostic methods that are sufficient to investigate the biochemical processes are helpful in the classification and treatment of pediatric brain tumors such as SPECT (single photon emission computed tomography) and MI (molecular imaging).

PET imaging may provide additional details for the structural lesions, especially non-enhancing tumors, such as low-grade gliomas [12], which may not be seen by computed tomography (CT). Gadolinium enhanced MRI is the therapeutic standard method of diagnosing brain tumors for adults and children. Natural and anomalous brain physiology can be analyzed in depth due to sensitization to various contrast parameters in MRI techniques [13]. In addition to offering a high spatial resolution, sagittal, coronal and axial direct multiplanar visualization along with an excellent soft tissue contrast [14], the major advantages of MRI include it being a non-invasive and painless operation.

The stored format of the MRI images can usually be categorized into two classes, the format of the scanner and the format of image processing. The scanner format is defined as the output of the computer that extracts MR images, and the other kind is defined as the image processing format created by the translation of the original format of the MRI scanner [15]. The magnetizing properties of the atomic nuclei are the foundation of the MRI. The application of additional radio frequency (RF) energy further disturbs this magnetization. Through various relaxation processes, the nuclei return to their resting magnetization and absorb radio frequency energy. The signals emitted are measured for a certain duration after the initial radio frequency. Various kinds of images are generated by adjusting the sequence of radio frequency pulses used and received. TE (the time to echo) is the time between the delivery of the RF pulse and the detection of the echo signal. TR (repetition time) is the amount of time between successive sets of pulses applied to the same slice [16]. Two separate T1 and T2 relaxation times can differentiate the tissue. T1 is the time constant that represents the rate at which the excited protons return to balance, while T2 is the time constant that defines the rate at which excited protons enter or exit the process in equilibrium with each other [16].

T1-weighted and T2-weighted scans are the most popular MRI sequences. Short TE and TR periods are used to produce T1-weighted images, while T2-weighted images are produced using longer TE and TR periods. Generally, by looking at the cerebrospinal fluid (CSF), the T1 and T2-weighted representations can be readily separated, while, in the T1-weighted imagery CSF is dark, the CFS in the T2-weighted imagery is bright. The FLAIR (fluid attenuated inversion recovery) sequence, which is another commonly used sequence, is comparable to a T2-weighted image, except that the TE and TR cycles are very long.

Despite the fact that MRI is the most successful choice for brain tumor diagnosis, detecting the degree and type of a tumor using traditional MRI is difficult [17]. Therefore, advanced MR techniques over traditional MRI, such as MRS (magnetic resonance spectroscopy), DWI (diffusion-weighted imaging), SWI (susceptibility-weighted imaging), PWI (perfusion weighted imaging), and DTI (diffusion tensor imaging) has provided significance to the evaluation of neoplastic histology, such as neovascularization, degree of cellularity, and mitotic index [18].

### 2.3. Reading MRI Sequences

**Figure 3:**
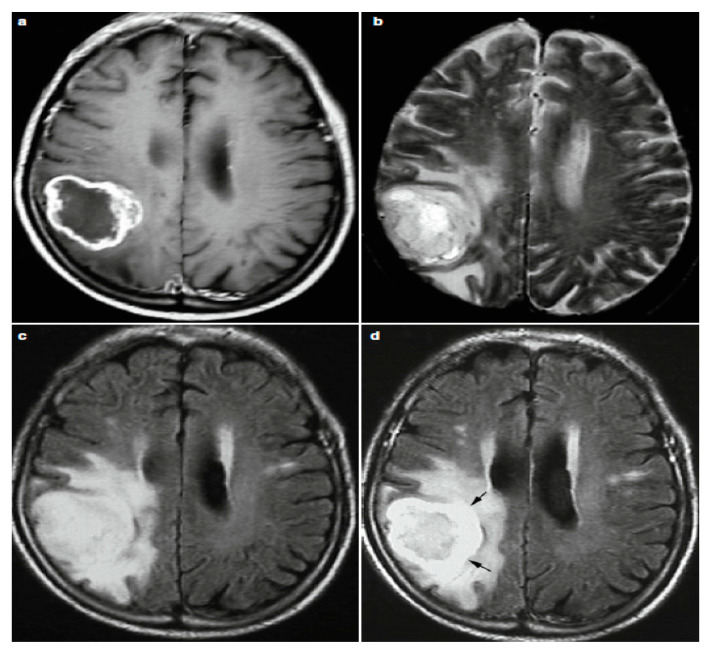
Four Different Image Modalities: (**a**) Post-Contrast T1w, (**b**) T2w, (**c**) FLAIR and (**d**) Post-Contrast FLAIR MRI.

Because of the growth and presence of the brain tumor, one MRI sequence is not enough to properly examine the tumor. Consequently, it is a time-consuming and complicated process for radiologists to analyze image characteristics and interpret MR images. In current clinical routine, various sequences of MRIs are used to diagnose and delineate tumor compartments [19]. Tumor orientation, heterogeneous intensity profiles, presence and overlapping intensity of the tumor tissue vary between these sequences, which can lead to several different diagnosis. It is a demanding task to distinguish between distinctive tumor types, each of which has the same features [20,21]. Some tumor types, such as glioblastomas, for instance, have blurry boundaries and are difficult to discern from healthy tissues. Therefore, T1, T2, T1c (T1 with contrast), PD (proton-density weighted), dMRI (diffusion magnetic resonance imaging) and FLAIR (fluid attenuated inversion recovery) sequences are required to better diagnosis. The comparison between these modalities literally provides individuality to each type of tissue [22] as shown in Figure 3.

In the contrast enhanced images T1-weighted (gadolinium—DTPA), as the frequently used sequence for structural analysis, the tumor boundaries look brighter because the contrast agent collects there due to destruction of the blood-brain barrier in the proliferative tumor zone. This is s that T1-weighted can easily realize the active tumor region whereas the region of the edema circling the tumor remains bright in the T2-weighted view. Another special sequence that tends to distinguish edema from cerebrospinal fluid (CSF) is T2 FLAIR [23]. Astrocytomas, for instance, are usually T1-w isointense and T2-w image hyperintense. While MRI rarely classify low-grade astrocytoma, most anaplastic astrocytoma enhances by contrast agents [13].

MRI, however, may show some non-specific results, such as T2-weighted hyper intensity and FLAIR, in pediatric brain tumor diagnosis, which may reduce diagnostic accuracy [24,25]. Nevertheless, an increase in contrast as seen in contrast enhancement MRI is a weak predictor for tumor size identification [26]. In fact, contrast enhancement represents the permeability of a weakened blood tumor barrier to both the vascular surface region and the contrast agent [26]. Additional diagnostic techniques capable of evaluating multiple metabolic processes, such as SPECT (computed tomography with single-photon emission), PET/CT (positron emission tomography) and MI (molecular imaging) are also effective in characterizing childhood tumors during diagnosis and follow-up after treatment [24,25].

In the clinical context, on the T2 and post-gadolinium and T1 images, the radiologist also manually defines the radiological concept of tumor boundaries by thresholding the borders between T2 and T1 contrast-enhanced lesions and the underlying normal tissues to measure the tumor’s outer boundaries.

### 2.4. Available Pediatric Brain Datasets

Successful training of artificial intelligence (AI) applications relies on massive, well labeled, balanced datasets [27]. A major obstacle to the development of high-quality image processing AI systems in radiology may be considered to be the development of these datasets, not just because the expense of generating these datasets is high, but also because access to current datasets is limited. Privacy issues about the exchange of patient data and the comparative benefit gained by medical AI companies from their own proprietary datasets are likely to hinder the sharing of these data. In order to address these issues, numerous initial releases from large public databases have been made available to researchers in recent years through several major ongoing projects across the world. Following are the available pediatric brain MRI datasets, which have been released for training and evaluation of brain tumor:

1. dHCP: The Developing Human Connectome Project (dHCP) [28] is an ERC-funded collaboration between King’s College London, Imperial College London and the University of Oxford. dBCP has two data releases as to date. The first open access data release consists of images of 40 representative term neonatal subjects. The imaging data includes structural imaging, structural connectivity data (diffusion MRI) and functional connectivity data (resting-state fMRI). The second open access data release consists of images of 558 neonatal subjects. The released dataset includes T1w and T2w structural data supplied as initial image data and after pipeline preprocessing. The images included in this release were obtained from infants born and imaged between 24–45 weeks of age. Using a dedicated neonatal imaging device which included a neonatal 32 channel phased array head coil, imaging was carried out on 3T Philips Achieva.
2. PBTA: Pediatric Brain Tumor Atlas (PBTA) [29] is a collaborative effort, which is led by the Children’s Brain Tumor Tissue Consortium (CBTTC), to accelerate discoveries for therapeutic intervention for children brain tumors diagnosed. The first release of the Pediatric Brain Tumor Atlas (PBTA) dataset, which comprises over 30 different types of pediatric brain tumors covering over 1000 subjects, occurred on September, 2018. Data types include match tumor/normal, whole genome data (WGS), RNAseq, proteomics, longitudinal clinical data, imaging data (including MRIs and radiology reports), histology slide images and pathology reports.
3. HCP: The Lifespan Human Connectome Project Development [30] lunch Lifespan HCP Release 1.0 in May 2019 for HCP-Development and HCP-Aging. All HCP-development (ages 5–21) data is shared in the NIMH Data Archive, NDA Collection. Lifespan HCP Release 1.0 data includes unprocessed data of all modalities (structural MRI, resting state fMRI, task fMRI, and diffusion MRI) for 655 HCP-D subjects, minimally preprocessed structural MRI data (only) for 84 subjects, and basic demographic data (age, sex, race/ethnicity, and handedness) for all released HCP-D subjects.
4. PING: Pediatric Imaging, Neurocognition, and Genetics [31] data of 1400 children aged between 3 and 20 years are included in this genetics data resource. PING data access is thoroughly handled by the NIMH Data Repository.
5. iSeg-2017 and iSeg-2019: Challenge data six-month infant brain MRI segmentation (iSeg-2017) [32]. Comparing (semi-)automatic algorithms for the segmentation of 6-month infant brain tissues and the calculation of corresponding structures was its goal of the iSeg-2017 competition. On a Siemens head-only 3 T scanner with a circular polarized head coil, all scans for the 10 infant subjects were obtained. The six-month infant brain MRI segmentation (iSeg-2017) [33] aims to facilitate automated six-month infant brain MRI segmentation algorithms from multiple sites. They offered iSeg-2017 data for training datasets. For the validation dataset, 13 T1 and T2 subject MR images are given. T1- and T2-weighted MR images from three different sites are used in the test dataset.
6. IBSR: Internet Brain Segmentation Repository [34]. Along with magnetic resonance brain image data, IBSR provides manually-guided expert segmentation results. Its aim is to promote the assessment and development of methods of segmentation. This dataset contains eighteen currently available subjects aged 7–71 years.
7. ABIDE I and ABIDE II: The first ABIDE [35] project launched in August 2012 reflects Autism Brain Imaging Data Sharing (ABIDE I). Seventeen foreign sites were interested in ABIDE I, exchanging previously acquired resting state functional magnetic resonance imaging (R-fMRI) data. ABIDE I is comprised of 1112 datasets, including 539 from ASD individuals and 573 from typical controls. ABIDE II [36]. In order to further encourage research on the brain connectome in ASD, ABIDE II was released in 2016. There are 19 sites in ABIDEII, donating a total of 1114 datasets from 521 ASD individuals and 593 typical controls.
8. CoRR: The Consortium for Reliability and Reproducibility [37]. The goal was to create an open science database for the imaging community to facilitate the assessment of the reliability and reproducibility of functional and structural connectomics studies. CoRR contains 33 datasets, 32 of which are available for download at present. Four of these datasets contains pediatric brain MIR images. IPCAS 2 includes 35 typically developing children. Each participant underwent two scanning sessions one month apart. Three modalities (T1/EPI (echo planar imaging)/DTI (diffusion tensor imaging)) of brain images were acquired for all subjects. IPCAS 7 includes 74 typically developing children. Each participant was scanned twice within a session. Three modalities (T1/T2/EPI) of brain images were acquired for all subjects.

### 2.5. Data Acquisition and Analysis Methods for Human Brain Activity

Biology and medicine data are not as direct and meaningful as physical signals. With the advancement in the technology, data from biological specimens could be captured directly or indirectly by sensors. The details derived from the data could then be used for analysis, diagnosis, and treatment. The method of sampling signals to calculate real-world physical conditions and converting the resulting samples into digital numeric values that can be manipulated by a computer is known as data acquisition. Whereas, the compilation and manipulation of data to generate useful results is referred to as data processing [38,39].

Electroencephalography (EEG) is one of the techniques for collecting data from the human brain. It was developed in the 1930s by Hans Berger, a German psychiatrist [40]. It is a noninvasive approach for detecting and recording brain electrical activity using electrodes connected to the scalp that track variations in electric potential on the skin surface caused by the activity of cerebral neurons and then amplify them to form a record (an encephalogram) [41]. Neurologists now use EEG to distinguish between functional and organic brain conditions, diagnose sleep disturbances, headaches, and to control brain activity throughout cardiac operations. The disadvantages of using an electroencephalograph in use include the equipment’s limited resolution and the ability to display and interpret data on a screen.

The second method is magnetoencelography (MEG). MEG is a technique for measuring the magnetic field generated by the human brain. It allows for a much higher spatial precision signal and interpretation over a much broader frequency spectrum than EEG. The behavior of the neuron population parallel to the scalp is much more receptive to the MEG signal than it is perpendicular. MEG is used in biomedical experiments to assess the roles of specific brain areas, as well as in clinical diagnostics and as a tool for locating abnormal regions during neurosurgical procedures [42,43,44].

The third type of magnetic resonance imaging is functional magnetic resonance imaging (fMRI), which detects an increase in blood supply and oxygenation in the active portion of the brain [45]. fMRI is focused on the application of magnetic resonance imaging (MRI) and its extension through observation based on the properties of oxygenated and deoxygenated blood [42]. The use of continuous magnetic field gradients to register these waves (caused by protons returning to their ground state emit an electromagnetic) allows a device to reconstruct the representation of the interior of the object under analysis [42].

Fourth, positron emission tomography (PET) is a procedure for imaging that records the radiation released during positron annihilation. The registered data is saved on a storage disk in digital form, allowing for the construction of cross-sectional photographs of the patient’s body, similar to those produced by MRI. Currently, almost all positron emission tomography scanners on the market are hybrid instruments of the type: PET-CT, PET/CT—PET with a multi-row computed tomography scanner PET-MRI, also known as PET/MRI, is a hybrid of PET and magnetic resonance imaging [42].

Finally, near infrared spectroscopy (NIRS) is a method for visualizing brain function that involves sending laser beams across the skull. Blood that has absorbed oxygen receives light waves at different frequencies than blood that has not absorbed oxygen. Researchers can monitor blood pressure by measuring the amount of light transmitted from the brain at different wavelengths. Diffuse optical tomography, or DOT, is the procedure used, if the purpose was to create the activation map. Whereas for registration purpose, it is an event based optical signal (EROS) that registers light diffusion due to shifts in cells that arise during the excitement of neurons. Although techniques like diffuse optical tomography and NIRS rely on blood flow to measure optical absorption of hemoglobin, EROS uses the scattering properties of neurons to provide a much more precise measure of cellular activity [42].

Theses modern data acquisition techniques focusing on brain impulses include EEG, NIRS, fMRI, and PET have a readout of the messages in the human brain, as well as a method for archiving and interpreting them as [43]. A multi-channel encephalograph was used to demonstrate the signal readout. Time-varying EEG signals from individual electrodes were captured in the. edf format using the Emotiv Xavier TestBench program, and then subjected to Toolbox EEGLab for Matlab.

The cerebral cortex’s pyramidal cells are thought to be the primary source of the electroencelographic signal in the human brain because of their unique position within the cerebral cortex structure [46,47]. It is frequently necessary to identify the signal source in the human brain and thereby isolate the interferences. There are a variety of methods for removing such artifacts, including blind signal separation, which separates unknown signals without knowing how they were mixed together [42]. Therefore, a number of studies have been carried out in order to pinpoint the location of generation electric activity in the human brain. Researchers attempting to ascertain the position of generation electric activity in the human brain as a source signal characteristic for a given neuron fraction have run into the problem of blind source separation, according to a recent study [48]. The sLORETA algorithm, which was also used to classify sources as part of the inverse problem, was provided along with a blind signal separation (BSS) technique with Moore-Penrose pseudo-inversion. Their findings indicate that, after blind source separation, Moore-Penrose pseudo-inversion works well for matrix generalization in the field of EEG signal reconstruction. The experiment, which used the sLORETA technique, proved that it is possible to observe changes in brain activity for specific mental tasks, allowing for the detection of the cause of a given potential. 

## 3. Pediatric Brain Tumor Deep Learning-Based Studies

**Figure 4:**
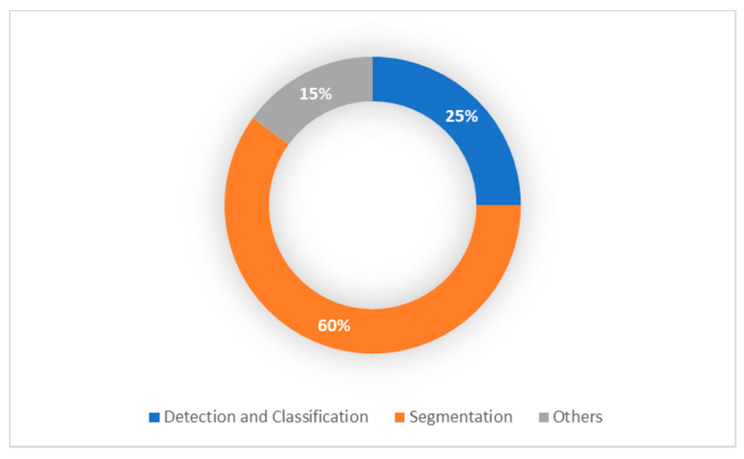
Pediatric Brain Tumor Deep Learning-Based Studies.

From 2015 onward, the topic of deep learning to brain tumor analysis has now become dominant at different conferences and journals. However, most of these studies have primarily been focused on data from adults. Whereas, few studies focused on children brain tumors. The advancement of pediatric brain tumor MRI techniques as well as the current success of the approaches of deep learning for brain tumor diagnosis inspired us to present a thorough overview of all pediatric brain tumor regions, including detection, classification and segmentation. As can be shown in Figure 4, most of the pediatric brain tumor studies focused on segmentation process due to the highly success in the segmentation process in adults MRI imaging.

### 3.1. Pediatric Brain Tumor Detection and Classification

Methods of deep learning have been used to detect and identify different brain abnormalities in children and fetals. This section presents existing methods for identifying and classifying pediatric brain tumor research based on deep learning. A thorough analysis of these studies can be found in the Table 1. 

Two early studies have demonstrated the capacity of neural networks to differentiate the major tumor types in the posterior fossa in children. In 1997, four neural networks were developed to incorporate MRS data with 10 tumor tissue characteristics obtained from magnetic resonance (MR) samples into patient tumor size, age and sex to increase diagnostic accuracy for 33 children in a dataset suffering from posterior fossa tumors [49]. The collected dataset was analyzed by a neuroradiologist, then the tumor types were divided into three categories on the basis of data acquired from MR imaging. Predictions were then compared with those generated by neural networks that evaluated different variations of data. Using multiple datasets as inputs, the four proposed neural networks were capable of correctly classifying the tumor type with 58% to 95%. They reported that the neural network, which was provided with imaging data, spectroscopic data and a limited amount of clinical information was able to accurately predict the type of pediatric posterior fossa tumor with exceptional precision. Their results also indicated that the predictive ability is improved with the increase of the input data size.

In the same context, in 2004, another neural network has been presented to classify the posterior fossa tumor [50]. Medulloblastoma, cerebellar astrocytoma and ependymoma tumors, from 33 pediatric patients, were analyzed and used for model training and testing. The proposed network was able to accurately identify 85.7% of the tumors when all the required information was available and only 72.7% in cases with incomplete information. They also stated that the diagnosis created by the network offered precise diagnoses in both cases that the neuroradiologist conducted.

For the four most popular pediatric posterior fossa tumor pathology identification and classification, a recent study established a deep learning model based on MRI [51]. Their dataset consisted of 617 children with four different types of posterior fossa tumors. As the basis of their multitask classifier model, they suggested a modified ResNeXt-50-32x4d architecture. With an F1 score of 0.80, the model classification accuracy exceeded 90% and the model tumor detection surpassed the area under the 0.99 ROC curve.

The authors used the innovative diffusion histology imaging (DHI) technique in another recent study [52] which incorporates deep neural networks and diffusion base spectrum imaging (DBSI). DHI is able to classify, differentiate, and measure heterogeneous regions of pediatric high-grade brain tumors. The proposed DHI (DBSI + DNN) approach could classify six distinct types of tumor histology components with an average precision of 83.3%.

A novel genomic algorithm (GA) defines optimum design parameters in order to classify adamantinomatous craniopharyngioma in children [53]. The efficiency enhancements for MRI-trained networks and 23% for CT-trained networks were reached by about 38% using GA as a meta-heuristic optimizer. This resulted in 85.3% test accuracy for computed tomography (CT), 83.3% for magnetic resonance imaging (MRI) and 87.8% for composite datasets of CT and MRI.

### 3.2. Pediatric Brain Tumor Segmentation

**Figure 5:**
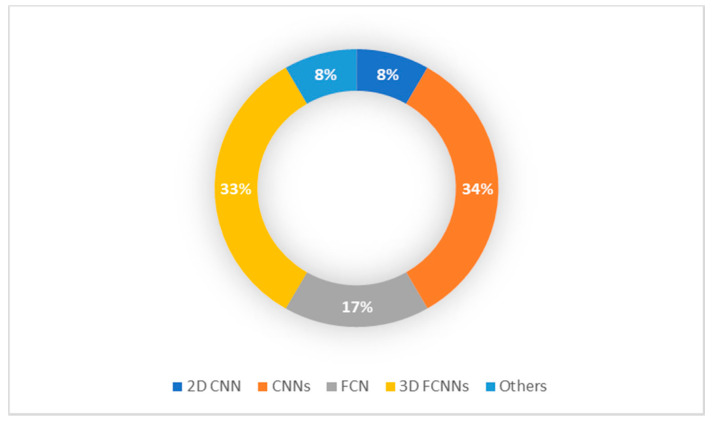
Pediatric Brain Tumor Segmentation Methodology.

For several years, brain MRI segmentation has become a growing field in computer vision. Segmentation is a fundamental phase in the quantitative study of brain imagery and the investigation of brain diseases. Most of the research, however, centered either on segmentation of adult tumors/disorder images or on normal brain segmentation for adults and/or children. As a result, there were a several studies, which have been conducted on pediatric brain tumor segmentation as summarized in Table 2. In addition, Figure 5 presents the most popular methodologies proposed in these studies.

All CNNs were proposed in 2015 using multi-modality MR images to segment isointense level brain tissues [54]. T1, T2, and FA (fractional anisotropy) multimodality images were used as input feature maps, and then segmentation maps were produced as output feature maps. The overall dice ratios total value over eight subjects achieved was 85.03%. Specifically, for the three types of brain tissues, dice ratios were generated by their proposed CNN on average over the eight subjects with 85.18% for GM, 86.37% for WM and 83.55% for CSF.

A new patch-based technique using a CNNs for automatic brain MRI segmentation was suggested by another study in 2016 [55]. Each brain MRI acquired from a public dataset is first subdivided into patches for this purpose. As a training input for the proposed CNNs, all of these patches are then utilized. They were able to segment over 90% of the brain MRI region with their convolutional neural networks. They reported that the 90% accuracy rate outperformed other traditional approaches and methods of machine learning. With only 100,000 patches that were only extracted in four brain MRIs and trained CNNs, complex edge pixels can be successfully segmented.

A novel method for the automated segmentation of anatomical MR brain images into a number of multi-scale CNN-dependent classes was developed in March, 2016 [56]. Their analyses demonstrate accurate segmentation effects in images acquired with varying acquisition procedures within different ages. Average dice coefficients for each of the five distinct datasets in all segmented tissue classes are as follows: 0.87 (coronal T2w 30 weeks), 0.82 (coronal T2w 40 weeks), 0.84 (axial T2w 40 weeks), 0.86 (axial T1w 70 years) and 0.91 (sagittal T1w 23 years).

A FCN was developed in 2016 in the form of segmentation of isointense phase brain MR images [57]. They operate a convolution-pooling stream for multi-modality data from T1, T2 and FA images. They then merge them into high-layer maps to generate segmentation maps effectively. For each single modality, they often implement the FCN architecture, and then present multi-FCNs (mFCNs) for multiple modalities to integrate their complementary information effectively. In general, mFCNs, especially in the segmentation of GM and CSF, have exceeded FCNs. The average dice ratios of 0.873 for GM and 0.887 for WM, 0.855 for CSF of eight subjects were obtained by mFCNs. On the other hand, FCNs met average dice ratios of 0.861 for GM, 0.885 for WM and 0.838 for CSF.

The authors consider integrating a neural network model with an iterative graphic optimization strategy in another study to restore pixel-wise segmentation of objects from an image database with sufficient bounding box annotations [58]. The suggested DeepCut model iteratively updates the set goals of the CNN model and utilizes a fully connected conditional random field (CRF) to regularize segmentation. The DeepCut model performs well in terms of accuracy relative to a model trained under complete supervision, and hence, greatly reduces the annotation effort required for analysis. The authors have also proposed various DeepCut models and associated them with a simplistic approach to weak supervision in CNN training. These models had overall mean in DSC (%) as follows: CNNnaïve (74.0), DCBB (86.6), DCPS (90.3), and CNNFS (94.1). An average DSC improvement of 12.6% for brain segmentation has also been reported.

A further model based on CNN, transfer learning and constructed 3D image formulation from 3D volumes was suggested in 2017 [59]. They simply stack successive 2D slices of a 3D volume in order to create a set of 2D “color” images; these 2D images reflect the input of a pre-trained FCN-based VGG network. On two types of brain MR images (MRBrainS13 and NeoBrainS12), the proposed model has been evaluated. The suggested model segment the neonatal brain precisely into various tissues on the NeoBrainS12 dataset. For all conducted experiments, their results are based on the dice coefficient: CoGM (0.79–0.87), BGT (0.89–0.93), UWM (0.91–0.95), BS (0.76–0.86), CB (0.91–0.94), Vent (0.85–0.88), and CSF (0.82–0.89). The suggested model ranked the second best of the 38 methods submitted for adults on the MRBrainS13 challenges. Their results based on the dice coefficient were GM (86.03), WM (89.29), and CSF (82.44) on T1 sequences whereas on T1, T1-IR, and FLAIR sequences they were GM (85.40), WM (88.98), and CSF (84.13).

A 3D semantic tissue segmentation model, which based on multi-stream FCNN and context-guided 3D, was developed to map all volumetric data directly to its volume-wise labels [60]. In conjunction, a multi-scale deep supervision has been developed to mitigating the possible gradient issue of disappearing during training. The average dice overlap coefficient (DOC) model validation on the iSeg-2017 dataset achieved: 0.916 for GM, 0.896 for WM and 0.954 for CSF.

In the context of the isointense phase of brain image segmentation, a multi-modality CC-3-D-FCN model was proposed in 2019 [61]. They integrate coarse layer information with a dense layer information in order to improve the segmentation efficacy of their model, and extra convolutions layers are also used to solve the bias of the signal problem. As reported, in terms of both segmentation accuracy and time cost, their proposed approach outperforms all comparable models on the same filed. Segmenting efficiency was obtained by CC-3-D-FCN in (DC) with: 0.9190 for WM, 0.9401 for GM and 0.9610 for CSF.

In 2019, a newly automated approach for segmenting brain tissue in fetal MRI into seven tissue classes using convolutional neural networks was introduced [41,62]. It was shown that by supplementing the training data with synthesized intensity inhomogeneity artifacts, the proposed approach learns to cope with intensity inhomogeneity artifacts. Their findings show that when the training data was enriched with simulated intensity inhomogeneity artifacts, the average achieved DC (dice coefficient) improved from 0.77 to 0.88, and MSD (mean surface distance) decreased from 0.78 mm to 0.37 mm across all tissue classes and images.

A FCNN that applies the dense connectivity principle to multi-modal segmentation problems (HyperDenseNet,) was developed in 2019 [63]. There are dense connections between pairs of layers along the same path and between pairs of layers around different pathways in each imaging modality. HyperDenseNet has been able to investigate diverse combinations of features of multiple modalities, inside and between all abstraction levels. A thorough analysis was applied to HyperDenseNet using MRBrainS for adult and iSEG-2017. HyperDenseNet outperforms baselines with a dice similarity coefficient (DSC) of 0.9580 for CSF, 0.9183 for WM and 0.9035 for GM. In the iSEG 2017 Challenge, their network ranked among the top-three models and ranked first in the MRBrainS Challenge, with the highest DSC and HD for GM and WM.

Authors focusing on an ensemble DCNNs for multimodality MRI for the isointense phase of brain image segmentation have introduced three different models [64]. Their study is the first to use an ensemble of three-dimensional convolutional neural networks to propose annotations within images. The way to measure the level of agreement of a group of predictors is a significant advantage. This is particularly useful for assessing the segmentation’s reliability at the voxel level and recommending local corrections in areas where the ensemble is uncertain about the prediction. Prediction uncertainty, measured as the opposite of predictor agreement within the ensemble, is strongly associated with segmentation errors, according to their findings. For this purpose, three different models have been implemented. The first method, called EarlyFusion-Single, is a semi dense network with an early fusion of multi-modal images. The second model, the EarlyFusion Ensemble, comprises a group of ten EarlyFusion CNNs trained in various subjects. The third model, the LateFusion Ensemble, is a set of ten semi dense CNNs, each conducting a late fusion of modalities in various paths and trained with distinct subjects. In the iSEG-2017 challenge, the success of the proposed solution was assessed. Their methods ranking first or second among the 21 participating teams for most of the metrics.

In this study [65], a novel method of enhanced transfer learning (TL) was suggested in this research to preserve generalization and reliability in the challenge of whole brain segmentation. With new datasets, they were able to improve the current whole brain segmentation algorithm SLANT (spatially localized atlas network tiles). They assume, however, that while the efficiency of the deep neural network can be increased with TL to accommodate certain dataset features, this will result in a decrease in the output of the actual training dataset. This assumption is assessed by a cohort of participants in pediatric study and a cohort of clinically obtained intravenous contrast data. Their results indicate that the original SLANT segmentation algorithm decreased pediatric brain output, presumably due to lower volume and altered proportions of gray/white matter in younger subjects relative to the initial training data used in SLANT. The T1w MRI with manually corrected volumetric labels is initially optimized for the age of thirty young children and the automated segmentation accuracy defined in relation to the manually assigned. The acquisition of thirty-six matched clinically acquired pre-contrast and post-contrast T1w MRI datasets and the accuracy of the measured post-contrast segmentation compared to the automatic pre-contrast evaluation was then optimized. SLANT has been improved with TL on all experiments. All methods achieved substantially better results over baseline SLANT (dice similarity coefficient (DSC): pediatric: 0.89; contrast: 0.80).

In [66], the authors evaluated both LiviaNET and HyperDense-Net models for neonatal brain imaging to segment neonatal brain tissue types at levels of equal age. The HyperDense-Net dual-modality achieved the highest mean test DSC values of the studied segmentation methods, reaching 0.94/0.95/0.92 for the types of tissue, as their results showed. For all tissue types, in the analysis of T2 weighted images the single modality LiviaNET, was higher than in the analysis of T1 weighted images with mean DSC values: 0.90 for WM, 0.90 for GM and 0.88 for CSF.

### 3.3. Related Pediatric Brain Tumor Studies

In addition to the classification and segmentation studies of brain cancer in children, some studies are specifically linked to these studies. In this section, we will list some of these studies that help to refine and enhance the study of children’s brain images. Table 3 shows these studies with some details.

Recent analysis has been undertaken in the field of attenuation correction, the initial purpose of it was to modify the current methodology of the RESOLUTE model [67] to the pediatric cohort model referred to as DeepUTE [68]. The RESOLUTE model is evaluated against the performance of a deep learning MR-AC approach. The proposed DeepUTE was the most equivalent, regardless of age, on the basis of both assessment metrics and visual inspection to obtain AC maps similar to CT-AC. Generally, DeepUTE outperformed RESOLUTE: for RESOLUTE/DeepUTE in Jaccard index: soft tissue 0.74/0.79, bone tissue 0.53/0.70 in bone tissue and for air 0.57/0.62.

**Table 3:** Related pediatric brain tumor deep learning-based studies.

Authors	Tumor Subject	Methodology	Modality	Dataset	Results
Ladefoged, Claes Nøhr, et al. (2018) [68]	Air, soft tissue and bone tissue	DeepUTE	PET/MRI (vendor-provided UTE images)	79 children (aged between 2–14 years)	Jaccard index 0.74/0.79 in soft tissue, 0.53/0.70 in bone tissue, 0.57/0.62 in air
Wang, Geliang, et al. (2020) [44]	Brain region volume Small-world properties Properties of brain structural network	BET, iBEAT and iBEAT with manual correction	3D T1WI	22 neonates (13 boys and 9 girls)	Brain regions analysis: significant differences in 50 brain region with iBEAT with manual correction showed the more accurate brain segmentation
Chang, Alex, et al. (2020) [69]	Whole body	DCGAN, StyleGAN, PGStyleGAN, StyleGAN2 + FID/DFD VAE for evaluation	360 wbMRI slices	90 healthy patients (ages 4 to 18)	FID, DFD, false positive rate: (457.30, 23.72, 0%) for DCGAN, (481.3, 19.378, 0%) for StyleGAN, (442.61, 18.56, 20%) for PGStyleGAN, (497.09, 17.234, 30%) for StyleGAN2

The influence of skull stripping on the neonate brain structural network’s size estimation has been quantified [44]. Compared to the 3D T1WI brain structural network, three tools including BET, iBEAT and iBEAT with manual correction were used to test the effect of skull stripping on the accuracy of segmentation of brain tissue and structural construction. However, a significant variation in brain volume and structural network property measures between the three tools, have been reported. The iBEAT with manual correction showed the more accurate brain segmentation, according to the results. 

Using GANs (generative adversarial networks) [69], authors demonstrated that GANs are capable of producing pediatrics wbMRIs required to allow automatic anomaly detection. In this study, samples generated using the StyleGAN2 architecture, in particular, had high visual quality, which the radiologist considered to be true. In order to identify tumor lesions, the role of anomaly detection using GAN trained on normal images was shown, that could minimize the need for limited examples of wbMRI tumors. They also argued that the FID (Frchet inception distance) metric is inadequate to compare image quality and that DFD (domain Frchet distance) metric is the suitable substitute. The results for each one of the GAN architectures are: DCGAN (457.30, 23.72, 0%), StyleGAN (481.3, 19.378, 0%), PGStyleGAN (442.61, 18.56, 20%), StyleGAN2 (497.09, 17.234, 30%) for FID, DFD and false positive rate for the radiologist blind test, respectively.

## 4. Medical and Technical Challenges

Medically, assessing brain tumors in children is a diagnostic concern due to various tumor pathology, non-specific or conflicting imaging results, recent evidence of gadolinium aggregation in the brain, susceptibility to near-skull tumor locations, and minimal signal-to-noise ratios. Early diagnosis of pediatric brain tumors relies almost entirely on the age of the patient, the place of the tumor and the reports of neuroimaging. Possible brain MRI objectives for pediatric brain tumors, in addition to early diagnosis, often cover separation between different types of tumor, tumor grading, distinguishing between active tumor and tissue damage, stereotactic biopsy guidance, and determination of treatment response. Advanced MRI techniques are commonly used in the MRI protocol, such as DWI, DTI, fMRI, MRS and SCEST, as traditional MRI is often incapable of achieving all objectives. Different difficulties have arisen in the implementation of deep learning methods for pediatric brain tumor image analysis as a consequence of discrepancies in current modalities, as well as the diagnostic challenges described above.

Treatment paradigms can range from single-modality therapy to variations of surgery, systemic therapy, targeted agents, and/or radiation therapy, depending on the clinical context of disease of each patient (e.g., histology, extent of disease, patient age). Significant advancements in neurosurgery, radiotherapy, and chemotherapy have resulted in increased recovery and cure rates for children with brain tumors in recent decades. 

In the past, surgery was the only available treatment option for pediatric brain tumors, and although many tumors cannot be removed via surgery by itself, surgery still plays a crucial role in treatment as it allows for the reduction of the tumor in size, which in turn may improve the treatment outcome. During the past century, radiotherapy has evolved as an accompanying treatment option, not only as adjuvant therapy for resected tumors, but also as a definitive treatment for unresectable tumors, as well as a prophylactic therapy for occult microscopic tumors. Both surgery and radiotherapy, however, pose an obstacle when it comes to the survival of pediatric brain tumors, due to their invasiveness and long-term CNS side effects, respectively [70].

For the wide range of lesions found, a number of surgical techniques are available. Surgical treatment can require biopsy for histological diagnosis, cytoreduction/debulking, and full excision for local oncological monitoring as well as treating complications like hydrocephalus and the installation of ventricular access devices (VADs) to allow intra-thecal/intraventricular adjuvant chemotherapy. In all of these cancers, the extent of resection (EOR) has a substantial impact on the oncologic outcome. Neurosurgical advancements, on the other hand, have concentrated on designing minimally invasive treatments that are as safe and cost-effective as open surgery, but with less patient pain and morbidity [71]. 

However, novel surgical strategies have been developed that help overcome the issue with poor survival when it comes to childhood brain tumors. One of those approaches is the minimally invasive laser-induced thermal therapy (LITT), which is based on delivering laser energy to the afflicted tissue directly via percutaneous insertion of an optical fiber, thereby destroying the afflicted tissue by inducing necrosis. This makes it highly suitable in cases where tumors arise in locations that are difficult to access with classical surgery, but also in cases where patients suffer from multiple recurrences as well as repeat resections [72,73].

Another novel method, stereotactic radiosurgery (SRS), which is also suitable for surgically inaccessible brain tumors, is based upon the delivery of a high and single radiation dose to a specific target, whereby it inhibits the growth of the tumor and is minimally invasive when it comes to the surrounding, unaffected tissue. The appeal of this method is due to its ability to combine the advantages of radiobiologic fractionation with radio surgical precision [74].

The shortage of large training datasets is, first and foremost, a significant challenge for deep learning approaches. Especially in pediatric applications, a high-quality labeled dataset available are particularly limited, since recruitment in such groups is considerably more difficult than in adults. The scarcity of such datasets has hindered the capability of deep learning to reach its maximum potential.

The dilemma of the class imbalance in medical applications is another significant issue. The problem of class imbalance has been reported to have a significant negative influence on the training of models of deep learning. Deep learning models that typically rely on large classes with imbalanced datasets, lead to low accuracy for a limited classis. In fact, an interpretation of how precise weights or inputs apply to the model’s final result is often difficult to measure. Such interpretations are incredibly important in order to effectively implement deep learning applications for early identification of deep learning approaches in a clinical environment.

Furthermore, in each image processing task, unique difficulties arise in particular. An example for this, it is the density of the various categories of tissue that is not consistent, but varies gradually across the space of the image which is cause significant barriers that prohibit segmentation in the MR images regardless of the applications available. Moreover, due to the higher frequency of motion artifacts when compared to adults, and lower contrast-to-noise ratios (CNR) due to the small size of the fetal/neonatal brain and shorter scanning times, fetal and neonatal brain segmentation is considerably more complex than adult brain segmentation. While in different tumor datasets, several existing methods of detection have been seen to achieve strong performance. A medical opinion is often needed for better diagnosis in all cases, irrespective of the accuracy percentages recorded by any tumor detection model. Ultimately, a significant hurdle to imaging methods is the computational difficulty of handling multi-modalities MR images.

The issues that should be addressed in the future include the proper manipulation of parameters and motion applied to images for accurate diagnosis during the MR image scan. Still, exclusive problems exist, in particular, in each mode of imaging, datasets, pathological environments, and testing experts need to be oriented in the near future to make them more cost-effective.

## 5. Conclusions and Future Directions

This review is to provide a detailed summary of the current state of pediatric brain tumors studies centered on medical imaging based on deep learning. Due to many challenges associated with this domain, there were relative scarcity of publications of deep learning-based studies of pediatric brain tumor images. Only a recent similar review study in this field was conducted a year ago [75], however, the emphasis was only on presenting deep teaching methods in infant MRI systems only in the segmentation of infant brain tissue at the isointense phase and pre-symptomatic condition predictive autism spectrum disorder (ASD).

For pediatric brain tumors, and before any treatment strategies is applied, it is important to consider the nature of the brain tumor, including its size, rate of growth. There are two main approaches to obtaining this information: surgery and imaging. Imaging approaches are favored for the diagnosis of disease, either before or after surgery, in terms of cost, risk and time considerations [76,77]. It is, however, sensitive to human subjectivity and, for human observation, a vast volume of data is challenging. The diagnosis of early brain-tumor mostly relies on the radiologist’s expertise [78]. Despite all the benefits offered by non-invasive imaging, it should be acknowledged that only after biopsy and histology will a definitive diagnosis be made. A biopsy is typically done to complete the diagnosis, in order to examine whether the tissue is benign or malignant. A biopsy of the brain tumor is typically not obtained until definitive brain surgery [79]. In general, biopsy diagnosis success rates are highest for tumor cases. The greatest risk, though, is bleeding from the biopsy needle in the tumor and brain that may cause anything from headache to stroke, coma, or even death [80].

Advanced MR techniques, such as MRS (magnetic resonance spectroscopy), DWI (diffusion-weighted imaging) and SWI (susceptibility-weighted imaging), PWI (perfusion weighted imaging), DTI (diffusion tensor imaging) have provided significance to the evaluation of neoplastic histology, such as neovascularization, degree of cellularity, and mitotic index [18]. As mentioned earlier, in Section 2.5, fMRI detects the increase in blood supply and oxygenation in the active portion of the brain [45]. It focuses on the application of magnetic resonance imaging (MRI) and its extension through observation based on the properties of oxygenated and deoxygenated blood [42]. A recent study showed that presurgical fMRI/ dMRI tractography in children with low-grade brain tumors is feasible and also plays a significant role in preoperative risk assessment and decision-making, neurosurgical preparation, and intraoperative tracking [81]. 

Although deep learning approaches have made considerable advances in medical imaging applications, certain issues remain unresolved and comparatively few approaches in the area of pediatric brain tumors have been used. The considerable variability of image appearance in scanning from newborns to 18-year-olds, as well as the low signal to noise environment, different image modalities (particularly MRI), display many difficulties in childhood due to inappropriate tissue appearance around the image. The relative lack of publications can be explained by these difficulties, on the one hand. On the other hand, for non-deep learning approaches, these challenges are difficult to solve, and the capability of deep learning probably enables researchers to address them.

In this study, the recent success of applying deep learning strategies to the pediatric brain tumor domain has been discussed. Despite the fact that deep learning models are especially successful, there are still open issues include datasets size limitations, class imbalance and the absence of interpretability.

In the near future, deep learning will have a tremendous opportunity to advance the quality and value of pediatric imaging. To reach this potential, pediatric radiologists need to overcome large hurdles, including the development of very diverse datasets and detailed labeling, many of which are specific to pediatric imaging indications. Therefore, by releasing many broad public databases in recent years, the medical imaging community has already begun to resolve this problem, for example the Lifespan Human Connectome Project Development (HCP) [33]. Therefore, deep learning will have full potential to both support and augment pediatric imaging.

Centered on the current literature on pediatric brain tumor strategies discussed in this manuscript, a variety of debates on improving the efficiency of the developed method can be inferred. The implementation of neural networks (NN) and its improved models has helped researchers a great deal. Many CNN architectures actually have several layers, such as batch normalization, and additional normalization layers. Moreover, using principles from optimization and probabilistic models, each architecture has been extremely advanced. By taking the computational advantage of handling small patches instead of the entire slice or volume, researchers in brain MR image analysis may train deep CNNs to achieve proper segmentation algorithms. The medical imaging community, which mainly uses shallow architectures, recognized this accomplishment overwhelmingly. Regardless of their architectures and results, the most proposed works listed in Table 2 used 2D FCNN. Efficient generalization requires an architecture of optimized layers that considers hyperparameters, correct training approaches and balancing classes for improved performance when operating on any model. In their respective implementations, approaches with a 2D CNN architecture with adequate depth [46,47], cascade [57], and parallel networks [49] demonstrated top efficiency from their results listed in Table 2.

Furthermore, with the rise of GANs (generative adversarial networks), GANs-based brain tumor experiments have seen promising progress in medical imaging studies, but few approaches have been used in children with MRI data. The power of GANs, however, lies in their ability to learn in an unsupervised and/or weakly-supervised manner. In particular, we see that the image-to-image translation accomplished by cGANs may have many other valuable uses in the medical imaging domain. For example, restoring the MR images acquired with certain items, such as motion, particularly in a pediatric environment, can help to reduce the number of repeated examinations. In this manuscript, we presented a study demonstrating the challenge of identifying abnormalities using GAN trained in healthy images to recognize tumor lesions, which may minimize the need for limited examples of wbMRI tumors [61].

The scene of pediatric brain tumor research programs has been fully updated by the self-learning potential of new deep learning techniques, for examples: three studies show significant results exceeded accuracy of 95% on posterior fossa tumor classification [41,42,43]. Although CNN’s success has been acknowledged, their full capacity in brain MRI research has not yet been fully leveraged. There is a persistent need for further research in this regard before the reliable CNN applications can be used for in medical clinics.

## Figures and Tables

**Table 1:** Pediatric brain tumor detection and classification studies based on deep learning.

Authors	Tumor Location/Type	Methodology	Modality	Dataset	Results
Arle, Jeffrey E., et al. (1997) [49]	Posterior fossa (astrocytomas, PNETs, and ependymoma)	Four back-propagation neural networks	MRS + MR + Metadata	Self-acquired dataset (33 children 6 months–14 years)	Classification accuracy rate 58–95%
Bidiwala, S. and Pittman (2004) [50]	Posterior fossa (astrocytom, ependymom, and medulloblastoma)	Neural networks	CT + MRI (T1WI, T2WI) + Metadata	Self-acquired dataset (33 Children)	Classification accuracy rate 72.7–85.7%
Quon, J.L., et al. (2020) [51]	Posterior fossa (diffuse midline glioma, medulloblastoma, pilocytic astrocytoma, and ependymoma)	Modified 2D ResNeXt-50-32x4d deep learning architecture	T2-weighted MRIs	Multi-institutional study (617 children)	Detection accuracy was AUROC of 0.99 Classification accuracy was 92%
Ye, Zezhong, et al. (2020) [52]	Several histologic elements of tumors of pediatric high-grade brain tumors	DHI model (DBSI + DNN)	Diffusion basis spectrum imaging (DBSI)	9 pediatric brain tumor post-mortem specimens	Overall classification accuracy rate—83.3%
Prince, Eric W., et al. (2020) [53]	Adamantinomatous craniopharyngioma	CNN + genetic algorithm as a meta-heuristic optimizer	CT + MRI + combined CT and MRI	Multi-institutional study (39 children)	Classification accuracies 85.3%, 83.3%, and 87.8%, in respect to modality.

**Table 2:** Pediatric brain tumor segmentation studies based on deep learning.

Authors	Segmented Subject	Methodology	Modality	Dataset	Results
Zhang, Wenlu, et al. (2015) [54]	Segmenting all three types of brain tissues (CSF, GM, WM)	Four 2D CNN	T1, T2, fractional anisotropy (FA) MRI	Self-acquired (10 infants, 6–8 months of age)	Overall dice ratios CFS 83.55% GM 85.18% WM 86.37%
Cui, Zhipeng, et al. (2016) [55]	Patch-based CNN segmentation of brain structure	Three different CN Ns	Manually segmented MRIs	Public dataset (CANDI neuroimaging access point 103 MRIs) small sets 4–5 MRI from each subject (6 to 17 year old age group)	Accuracy rate of 90%
Moeskops, Pim, et al. (2016) [56]	8 subjects: CB, mWM, BGT, vCSF, uWM, BS, cGM, and eCSF.	CNNs	T1-weighted and T2-weighted MRI	Self-acquired (10 images at 30 weeks, 12 images at 40 weeks, 15 images at 23 years, 20 images at 70 years)	Average dice ratios 0.87 (coronal T2w 30 weeks), 0.82 (coronal T2w 40 weeks), 0.84 (axial T2w 40 weeks), 0.86 (axial T1w 70 years) and 0.91 (sagittal T1w 23 years).
Nie, Dong, et al. (2016) [57]	Segmenting all three types of brain tissues (CSF, GM, WM)	FCNs + multi-FCNs (mFCNs)	T1, T2, fractional anisotropy (FA) MRI	Self-acquired 10 healthy infants (6–8 months of age)	Average dice ratios FCNs (0.838 for CSF 0.861 for GM 0.885 for WM) mFCNs (0.855 for CSF 0.873 for GM 0.887 for WM)
Rajchl, Martin, et al. (2016) [58]	Whole brain pixel-wise segmentation	CNNs + fully connected conditional random field (CRF)	T2-weighted ssFSE sequence	Public dataset (55 fetal MRI subject)	DSC (%) CNNnaïve (74.0), DCBB (86.6), DCPS (90.3), CNNFS (94.1)
Xu, Yongchao, et al. (2017) [59]	Neonatal (CoGM, BGT, UWM, BS, CB, Vent, CSF) adults (CSF, WM, GM)	FCN + TL (VGG 16 network)	T1, T1-IR, FLAIR MRI	NeoBrainS12 + MRBrainS13	Dice coefficient Neonatal: CoGM (0.79–0.87), BGT (0.89–0.93), UWM (0.91–0.95), BS (0.76–0.86), CB (0.91–0.94), Vent (0.85–0.88), CSF (0.82–0.89) Adults GM (85.40), WM (88.98), CSF (84.13)
Zeng, Guodong, and Guoyan Zheng (2018) [60]	Segment isointense infant brain MRI (CSF, GM, WM)	3D FCNNs	T1 and T2 weighted MRI	Public dataset (MICCAI iSEG-2017)	Dice overlap coefficient CSF (0.954), GM (0.916), WM (0.896)
Nie, Dong, et al. (2019) [61]	Segment isointense infant brain MRI (CSF, GM, WM)	3D FCNNs	T1, T2, fractional anisotropy (FA) MRI	Self-acquired (11 healthy infants MRIs)	Dice ratios 0.9190 for WM, 0.9401 for GM, 0.9610 for CSF
Khalili, Nadieh, et al. (2019) [62]	Segment of seven brain tissue classes: cerebellum, basal ganglia and thalami, ventricular cerebrospinal fluid, white matter, brain stem, cortical gray matter and extracerebral cerebrospinal fluid.	2D FCN with identical U-net architecture	T2-weighted MRI	Self-acquired 12 fetuses (22.9–34.6 weeks post menstrual age) + neonatal MRI (40 weeks of post menstrual age) From the NeoBrainS12 dataset	DC over all tissue classes increases to 0.88 and MSD decrease to 0.37 mm
Dolz, Jose, et al. (2019) [63]	Segmenting all three types of brain tissues (CSF, GM, WM)	3D FCNNs	Integrated T1 and T2 MRI	iSEG-2017 + MRBrainS-2013	Baselines results with DSC (CSF 0.9580, WM 0.9183 and GM 0.9035)
Dolz, Jose, et al. (2020) [64]	Segment isointense infant brain MRI (CSF, GM, WM)	3D FCNNs	T1-weighted and T2-weighted MRI	Public dataset (MICCAI iSEG-2017)	Accuracy rate 92–96% Ranked as first or second in most metrics in the MICCAI iSEG-2017 challenge
Bermudez, Camilo, et al. (2020) [65]	Whole brain segmentation	SLANT + TL	T1-weighted brain MRI with and without intravenous contrast	Public dataset—Open Access Series on Imaging Studies (OASIS) 45 subjects aged 18–96 years old, 30 pediatric subjects (aged 2.34–4.31 years old) 36 subjects paired	DSC Pediatric: 0.89 Contrast: 0.80.
Ding, Yang, et al. (2020) [66]	Three types of brain tissues (CSF, GM, WM)	LiviaNET and HyperDense-Net CNNs architectures	T1-weighted and T2-weighted MRI	Publicly dataset DHCP (Developing Human Connectome Project), 40 healthy neonates born	Dual-modality HyperDense-Net accuracy rate: 92–95% Single-modality LiviaNET accuracy rate: 88–90%

## Data Availability

Not applicable.
